# Introducing Trifluoromethyl to Strengthen Hydrogen Bond for High Efficiency Organic Solar Cells

**DOI:** 10.3389/fchem.2020.00190

**Published:** 2020-03-24

**Authors:** Hao Zhang, Xiaoyang Du, Yunhan Tang, Xi Lu, Lei Zhou, Caijun Zheng, Hui Lin, Silu Tao

**Affiliations:** School of Optoelectronic Science and Engineering, University of Electronic Science and Technology of China (UESTC), Chengdu, China

**Keywords:** organic solar cells, ternary devices, hydrogen bond, π-π stacking, energy transfer

## Abstract

Nowadays, the ternary strategy has become a common way to improve the power conversion efficiency (PCE) of organic solar cells (OSCs). The intermolecular interaction between the third component and donor or acceptor plays a key role in achieving a high performance. However, hydrogen bond as a strong intermolecular interaction is rarely considered in ternary OSCs. In this work, we introduce trifluoromethyl on a newly synthesized small molecular DTBO to strength hydrogen bonds between DTBO and IEICO-4F. Due to the existence of hydrogen bonds has a strong impact on electrostatic potential (ESP) and benefits π-π stacking in the active layer, the ternary OSCs show superior charge extraction and low charge recombination. In DTBO, PTB7-Th and IEICO-4F based ternary devices, the PCE increases from 11.02 to 12.48%, and short-circuit current density (*J*_*SC*_) increases from 24.94 to 26.43 mA/cm^2^ compared with typical binary devices. Moreover, the addition of DTBO can realize an energy transfer from DTBO to PTB7-Th and broaden the absorption spectrum of blend films. Grazing-incidence wide-angle X-ray scattering (GIWAXS) patterns show that the π-π stacking distance of IEICO-4F decreased after adding 10 wt% DTBO. The effect of the hydrogen bond is also achieved in the PM6: Y6 system, showing 16.64% efficiency by comparison to the 15.49% efficiency of binary system. This work demonstrates that introduce trifluoromethyl to enhance hydrogen bond which improve π-π stacking can achieve higher performance in OSCs.

## Introduction

Solar energy is a promising alternative energy for future renewable energy. OSCs are the research focus in photovoltaic industry due to their superiority of low cost, flexibility and lightweight (Rossander et al., 2017; Dong et al., 2019; Jeong et al., 2019; Yan et al., 2019). Synthesis of novel acceptors has contributed to increase the power conversion efficiency (PCE). The state-of-the-art binary OSCs with single-junction structure have made a great improvement in PCE, which is up to 15% (Yuan et al., 2019). Nevertheless, it is a great challenge to further improve PCE. Many researchers have made much effort and got outcomes by adding solvent additives (Lee et al., 2008; Liang et al., 2010; Moon et al., 2010), incorporating the third material in the active layer (Bi et al., 2018; Nian et al., 2018), designing new device structure (Meng et al., 2018) and interface engineering (He et al., 2015). To date, ternary OSCs (containing a second donor or a second acceptor) has become one of the main strategies to elevate the PCE (Bi and Hao, 2019), the highest PCE of ternary devices is over 16% (Pan et al., 2019; Yan et al., 2019; Du et al., 2020).

In OSCs, π-π stacking plays a key role in device performance, which is extremely important for the charge transfer and transport (Ran et al., 2017; Hou et al., 2018). In addition, molecular aggregation is crucial to the exciton dissociation efficiency, high charge extraction, and low charge carrier recombination, leading to a better carrier mobility and domain purity (Zhang et al., 2017, 2018). Hence, incorporating a third component can achieve a better impact on π-π stacking and molecular aggregation, resulting in a better charge carrier transport channel and a better device performance.

To date, non-fullerene acceptors have shown remarkable performance. But one of the main challenges for non-fullerene acceptor is how to maintain efficient π-π interactions (Kang et al., 2016; Lee et al., 2016; Hou et al., 2018). Therefore, non-fullerene acceptors are the perfect materials to study the impact of the third component on the π-π stacking. Meanwhile, in ternary OSCs, incorporating a third material can broaden the absorption spectrum, resulting in enhanced photon harvesting ability and increased short-circuit current density (*J*_*SC*_) (Ameri et al., 2013; Yao et al., 2017; Jiang et al., 2018). To date, OSCs with very common donor PTB7-Th and narrow band gap non-fullerene acceptor IEICO-4F can easily achieve a high *J*_*SC*_ over 25 mA/cm^2^ (Song et al., 2019; Zhu et al., 2019). Since the fluorine atom has a strong electronegative property which can easily form hydrogen bonds with N-H group, IEICO-4F is an ideal non-fullerene acceptor to study the impact of hydrogen bonds on π-π stacking and obtain a good performance.

In this work, we designed and synthesized a novel small molecule with a simple structure, DTBO (7-(dibutylamino)-3-(6-(trifluoromethyl)-1H-benzo[d]imidazol-2-yl)-2H-chromen-2-one), via introducing trifluoromethyl on a previously reported small molecule Coumarin 7 (3-(1H-benzo[d]imidazol-2-yl)-7-(diethylamino)-2H-chromen-2-one) (Du et al., 2018). Due to the poor performance in ternary non-fullerene OSCs, we introduce trifluoromethyl on Coumarin 7 to strengthen the hydrogen bond with the non-fullerene acceptor in order to obtain a great impact on the acceptor. This small molecule was added to a PTB7-Th: IEICO-4F binary system as a nonvolatile additive to fabricate ternary OSCs. Because of strong electronegative of F atom in IEICO-4F, DTBO can easily form a hydrogen bond with IEICO-4F, which can be testified by Fourier transform infrared (FT-IR). Due to strong electron pulling effect of trifluoromethyl, theoretical predictions demonstrate that the hydrogen bond between DTBO and IEICO-4F is much stronger than that between Coumarin 7 and IEICO-4F. The existence of hydrogen bonds enhances the electrostatic potential (ESP) of IEICO-4F which promotes the charge transfer between the donor and acceptor. The intermolecular interactions between DTBO and IEICO-4F decreases the π-π stacking distance, leading to significant improvement charge extraction, and low charge recombination in comparison to the binary film. Moreover, DTBO has complementary absorption spectrum compared with PTB7-Th and IEICO-4F, which enhances photon harvesting ability of ternary blend. As a result, after adding 10 wt% DTBO, PCE, FF, and *J*_*SC*_ of the PTB7-Th: IEICO-4F based OSCs increased from 11.02 to 12.48%, 64.97 to 67.64% and 24.94 to 26.43 mA/cm^2^, respectively. On the contrary, owing to weak hydrogen bonds between Coumarin 7 and IEICO-4F, the performance of the ternary device is not as good as DTBO. This result suggests us that enhance intermolecular interactions is an effective way to achieve a high PCE. Recently, many non-fullerene acceptors have shown excellent performance. To further prove this strategy's universality in other non-fullerene acceptors which have an end group like that of IEICO-4F, we also use PM6: Y6 as the host system to fabricate ternary OSCs with DTBO, and the final PCE improved from 15.49 to 16.64%.

## Results and Discussion

### Characterization

**Figure 1:**
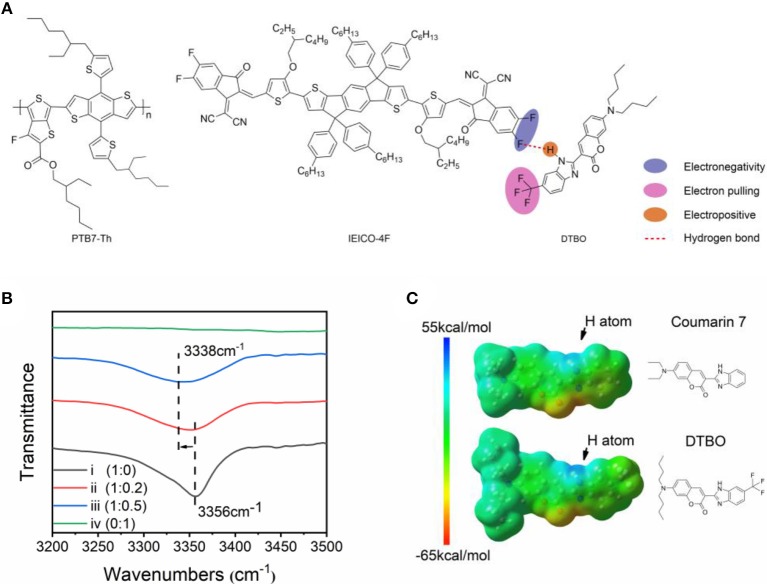
**(A)** Chemical structure of PTB7-Th, IEICO-4F and DTBO; **(B)** FT-IR spectra of DTBO: IEICO-4F blend films with different molar ratios: (i) DTBO: IEICO-4F = 1: 0, (ii) DTBO: IEICO-4F = 1: 0.2, (iii) DTBO: IEICO-4F = 1: 0.5, (iv) DTBO: IEICO-4F = 0: 1; **(C)** ESP maps of DTBO and Coumarin 7.

The chemical structures of DTBO, PTB7-Th, IEICO-4F are shown in Figure 1A. The synthetic procedures and synthetic scheme of DTBO are shown in the Supporting Information. The 1H NMR spectra, 13C NMR spectra and IR spectra are shown in Figures S10–S13. Since fluorine is the most electronegative atom in the periodic table of the elements, the N-H group in Coumarin 7 can easily form a hydrogen bond with the fluorine atom in IEICO-4F. In order to strengthen the hydrogen bond between DTBO and IEICO-4F, trifluoromethyl was introduced into DTBO. Due to strong electron pulling effect of trifluoromethyl, the hydrogen atom in N-H group shows higher ESP than Coumarin 7 which benefits hydrogen bond strength.

Fourier Transform infrared spectroscopy (FT-IR) was used to determine whether hydrogen bonds formed (Du et al., 2019). In Figure 1B, the pure DTBO film has a sharp wave trough at 3,356 cm^−1^ which is the characteristic wave trough of N-H bond, when adding different molar ratios of IEICO-4F the wave trough gradually moved to 3,338 cm^−1^. For the reason that there is no N-H group in IEICO-4F, IEICO-4F does not show any wave trough at 3,000 to 3,500 cm^−1^. As we known, the wave trough of N-H group will move to small wavenumber when a hydrogen bond is formed. The shift of FT-IR implied that the intermolecular hydrogen bonds existed (Steiner, 2003).

Hydrogen bond is essentially electrostatic interactions. Therefore, the molecular electrostatic potential was calculated by density functional theory (DFT) with the B3LYP/6-31G (d, p) basis set, based on the ground state geometries of Coumarin 7, DTBO and IEICO-4F. The calculations were performed by Gaussian 09 and the ESP statistics of each atom were conducted by Multifwn (Lu and Chen, 2012) The ESP distribution of DTBO and Coumarin 7 are illustrated in Figure 1C, the atom labels in these two molecules are shown in Figure S1, and the average ESP values on the atoms were calculated and summarized in Figure S2. The result suggests that the surface of hydrogen atom in N-H group shows strong electropositive. As for DTBO, the incorporation of trifluoromethyl group shows notable influence on the ESP. In N-H group of DTBO and Coumarin 7, the ESP of DTBO around the hydrogen atom increases to 30.99 kcal/mol compared with Coumarin 7 (26.57 kcal/mol), the improvement of ESP will enhance the strength of hydrogen bond. Furthermore, we calculated the binding energy of hydrogen bond, the binding energy between DTBO and IEICO-4F (6.40 kcal/mol) is higher than that between Coumarin 7 and IEICO-4F (5.57 kcal/mol), which means DTBO and IEICO-4F have much more stable intermolecular interaction.

**Figure 2:**
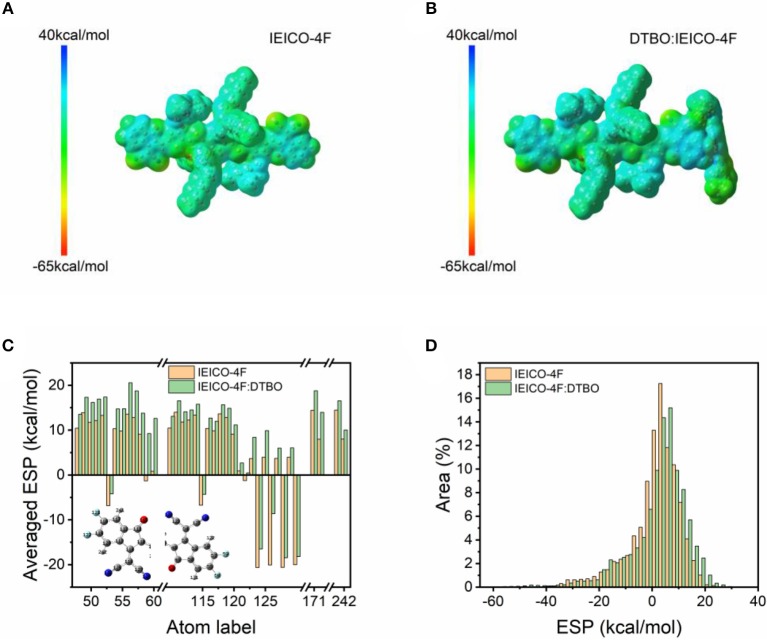
ESP maps of **(A)** IEICO-4F and **(B)** hydrogen-bond-linked IEICO-4F: DTBO; **(C)** Average ESP statistics of each atom in end group; **(D)** ESP area distributions of the molecules.

To further clarify the influence of intermolecular hydrogen bonds on charge distribution, the ESP maps of hydrogen bond linked IEICO-4F: Coumarin 7 system and IEICO-4F: DTBO system were calculated respectively. Figures 2A,B and Figure S3A are the ESP maps of IEICO-4F and hydrogen-bond-linked IEICO-4F, Figures S3B–D are the ESP area distributions. As shown in Figure 2D, for the IEICO-4F, the proportion of positive surface area is 67.89%, the maximal value of ESP is 24.26 kcal/mol. Notably, the proportion of positive surface area increased to 70.59% in hydrogen-bond-linked IEICO-4F: Coumarin 7 system, the maximal value of ESP increased to 30.14 kcal/mol. For the hydrogen-bond-linked IEICO-4F: DTBO system, the proportion of positive surface area did not change too much (70.91%), but the maximal value of ESP kept growing to 38.09 kcal/mol, which means the electrostatic attraction between the hydrogen-bond-linked IEICO-4F and PTB7-Th will be stronger than that between pure IEICO-4F and PTB7-Th. According to pervious report, acceptor has high and positive ESP on the most part of its surface while donor has negative ESP. The different ESP can produce an intermolecular electric field (IEF) between acceptor and donor, which facilitates the charge generation (Yao et al., 2018, 2019). In no-fullerene acceptor, the electron deficient end-capping units forms π-π interactions with the polymer donor in the blend film. As shown in Figure 2C, the existence of hydrogen bond increases the ESP of end group in IEICO-4F. As a result, the larger difference of ESP between IEICO-4F and PTB7-Th will induce a larger IEF, the larger IEF at the D-A interface facilitates the charge transfer more efficiency. Our results show that hydrogen-bond-linked IEICO-4F has a higher proportion of positive surface area, the donor and acceptor would show a larger potential difference in the ternary OSC, which is beneficial to charge generation and transfer.

**Figure 3:**
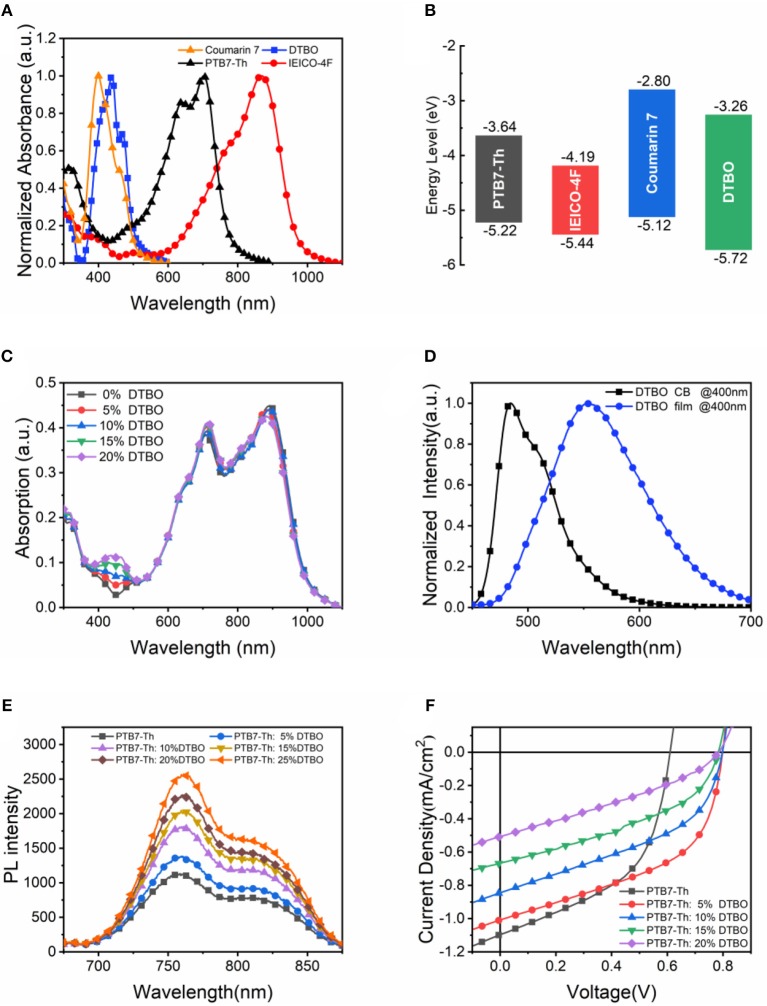
**(A)** UV vis absorption spectra of neat films; **(B)** HOMO and LUMO energy levels; **(C)** Absorption spectra of ternary films with different DTBO content; **(D)** PL spectra of DTBO in chlorobenzene solution and neat DTBO film under 400 nm light excitation; **(E)** PL spectra of PTB7-Th and DTBO blend films under 450 nm light excitation; **(F)** *J-V* curves of PTB7-Th and DTBO based devices.

The UV vis absorption spectra of neat films are shown in Figure 3A. The highest occupied molecular orbital (HOMO) and lowest unoccupied molecular orbital (LUMO) energy levels are shown in Figure 3B. The absorption peak of Coumarin 7 is at 398 nm, while the DTBO has a red-shift of ~40 nm corresponding to an optical band gap (*Eopt g*) of 2.46 eV. From the cyclic voltammetry (CV) plots (Figure S5), we can determine that the HOMO level and the LUMO are down-shifted for DTBO compared to the values for Coumarin 7, which is consistent with the theoretical calculation (Figure S4). Since the absorption band of DTBO is located in the short-wave region and PTB7-Th: IEICO-4F have strong absorption in the long-wave region, the ternary system absorbs more solar photons than binary system, resulting in an increased *J*_*SC*_ (Jiang et al., 2018).

The absorption spectra of blend films with different DTBO content were measured and shown in Figure 3C. The weight ratio of PTB7-Th and IEICO-4F is 1: 1.5, and DTBO was added to the binary system as the third compound with different PTB7-Th: DTBO weight ratios. Obviously, the absorption from 400 to 500 nm enhanced by introducing DTBO into the binary films. Because of the enhancement of absorption, a moderate amount of DTBO can boost the photon harvesting. In addition, there is a little increase in absorption spectra from 600 to 900 nm when blending DTBO, suggesting that the crystallization and arrangement of donor and accepter were changed.

According to the previous research (Yang et al., 2013; Gupta et al., 2015; An et al., 2016; Li et al., 2018), Förster-type energy transfer occurs when the absorption spectrum of one compound overlaps the emission spectrum of another compound. Figure 3D shows photoluminescence (PL) spectrum of neat DTBO film. It shows that the PTB7-Th has a strong absorption from 550 to 750 nm, and the maximum PL emission peak of DTBO is located at 553 nm, therefore the ternary films have the basic conditions for the energy transfer. In Figure 3E, as the content of DTBO increases gradually, the PL intensity of PTB7-Th: DTBO films gradually increases, implying the energy transfer from DTBO to PTB7-Th (Gao et al., 2020; Ma et al., 2020a). This phenomenon will boost PTB7-Th to produce more excitons which dissociate at the PTB7-Th: IEICO-4F interfaces, resulting an increased *J*_*SC*_ (Zhao F. et al., 2017). To further explore the effect of this small molecule on PTB7-Th, devices with only DTBO and PTB7-Th were fabricated to investigate the charge transfer or exciton dissociation between PTB7-Th and DTBO. The *J-V* curves were measured under AM 1.5G illumination with light intensity of 100 mW/cm^2^ (Figure 3F). The *J*_*SC*_ of DTBO based device is <4 × 10^−6^ mA/cm^2^. The *J*_*SC*_ of DTBO and PTB7-Th based devices is between DTBO based devices and PTB7-Th based devices. This result shows us that there is no charge transfer or exciton dissociation at DTBO/PTB7-Th interfaces. Based the analysis above, the exciton generated by DTBO transfer its energy to PTB7-Th by energy transfer process and then dissociated at PTB7-Th/IEICO-4F interface. The photon-generated carriers will be transported to electrode through the channel formed by IEICO-4F or PTB7-Th induced increased *J*_*SC*_ in ternary devices.

**Figure 4:**
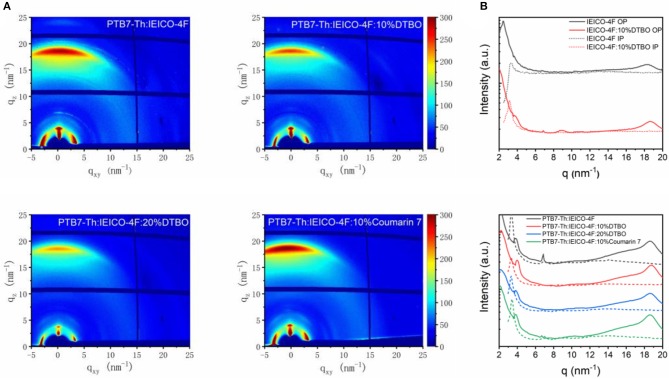
**(A)** 2-D GIWAXS X-ray scattering pattern; **(B)** line-cut profiles of binary and ternary films.

Since DTBO can change the ESP of IEICO-4F, it is reasonable to use GIWAXS to check the impact of DTBO on the molecular packing and crystallization of donor and accepter (Collins et al., 2012; Tumbleston et al., 2014). The 2-D diffraction patterns and line-cut profiles are shown in Figure 4 and Figure S6. Obviously, the molecular packing of both binary and ternary samples show a favored face-on orientation relative to the substrate, and exhibit strong π-π stacking signal in the out-of-plane (OP) direction (Rivnay et al., 2012). To further analyze the details of line cuts from the GIWAXS patterns, peak fit analysis was used in this study (Liu et al., 2014; Zhao et al., 2016). As seen from Figure 4B, pure IEICO-4F showed a strong (010) π-π stacking at about 18.28 nm^−1^ in the OP direction. When blended with 10% DTBO, the (010) diffraction peak shifted from 18.28 to 18.64 nm^−1^ and the d-spacing changed from 3.44 to 3.37 Å. As for pure PTB7-Th (Figure S6C), the OP (010) π-π stacking peak is observed at 16.33 nm^−1^. However, the position of (010) π-π stacking peak in PTB7-Th: 10% DTBO (16.33 nm^−1^) film did not change. This phenomenon may cause by intermolecular interactions between DTBO and IEICO-4F.

The coherence length (L_C_) was calculated by Scherrer equation from the full width at half-maximum (FWHM) of OP π-π stacking peaks. For the PTB7-Th: IEICO-4F binary film at high q region, according to peak fitting analysis, the blend film exhibits two diffraction peaks at 17.15 nm^−1^ (PTB7-Th), 18.66 nm^−1^ (IEICO-4F), and the L_C_ of PTB7-Th and IEICO-4F are 1.25 nm, and 4.60 nm, respectively. With addition of 10% DTBO, the diffraction peaks of PTB7-Th (17.17 nm^−1^) did not change, while the peak of IEICO-4F changed to 18.81 nm^−1^. As a result, the d-spacing of IEICO-4F changed from 3.37 to 3.34 Å. The level of crystallinity along OP direction influences the charge transport of the blend film. The crystallinity of IEICO-4F along OP direction in ternary film slightly decreased from 4.60 to 4.49 nm when adding 10% DTBO which is harmful in devices. But the *J*_*SC*_ of 10% addition ternary devices increased may contribute by the decreased of d-spacing (Mukherjee et al., 2016; Ran et al., 2018) When adding more DTBO (20%) in the ternary blend, the d-spacing of PTB7-Th (3.70 Å) and the L_C_ of IEICO-4F (4.15 nm) are reduced a lot compared with binary and ternary (10% DTBO) films. π-π stacking is vital to the charge transport, the decreased d-spacing and similar L_C_ indicate that the addition of 10% DTBO is an optimal choice. Just like ternary blend with 20% DTBO, the ternary blend with 10% Coumarin 7 show increased d-spacing and reduced L_C_ compared with 10% DTBO, indicating that addition of Coumarin 7 is not good as the 10% DTBO ternary blend.

### Device Performance

**Figure 5:**
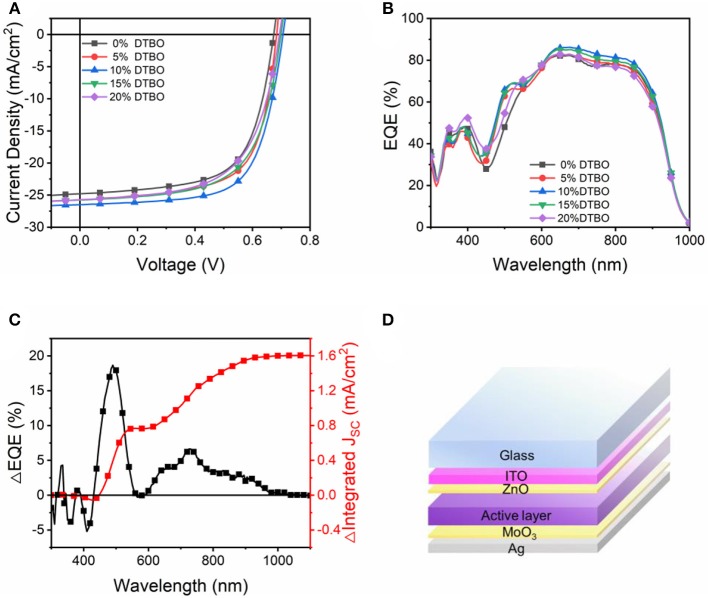
**(A)** *J–V* curve of OSCs devices; **(B)** EQE curves of the devices with different DTBO content in the PTB7-Th: IEICO-4F systems; **(C)** ΔEQE for 10% DTBO ternary blends; **(D)** Inverted OSCs architectures used in this study.

In order to prove the previous analysis is correct, ternary solar cells were fabricated with inverted (ITO/ZnO/active layer/MoO_3_/Ag) architectures (Figure 5D). The weight ratio of PTB7-Th to IEICO-4F was kept at 1:1.5 (8 mg/ml for PTB7-Th), and PTB7-Th: DTBO weight ratios are 1:0, 1:0.05, 1:0.1, 1:0.15, and 1:0.2 in this study. Each active layer thickness was maintained at ≈100 nm. Typical *J-V* curves of inverted ternary OSCs are illustrated in Figure 5A, with the corresponding PCE metrics summarized in Table 1.

**Table 1:** The photovoltaic parameters for binary and ternary OSCs under AM 1.5 G illumination (100 mW/cm^2^).

**Third compound**	***V_***oc***_*^**a**^ (V)**	***J_***sc***_*^**a**^ (mA/cm^**2**^)**	***J_***calc***_*^**b**^ (mA/cm^**2**^)**	**FF^**a**^ (%)**	**PCE^**a**^^,^^**c**^ (%)**
0% DTBO	0.68	24.94	24.06	64.97	11.02 (11.39)
5% DTBO	0.69	25.66	24.48	66.08	11.65 (12.15)
10% DTBO	0.70	26.43	25.66	67.64	12.48 (12.88)
15% DTBO	0.69	25.78	25.29	63.90	11.30 (11.66)
20% DTBO	0.70	25.86	24.51	61.60	11.10 (11.41)
10% Coumarin 7	0.69	26.25	24.91	66.53	12.06 (12.33)

a *All average values were calculated from 10 devices*.

b *J_sc_integrated from the EQE spectrum*.

c *Best PCE in brackets*.

The PTB7-Th: IEICO-4F binary control devices exhibit an average PCE of 11.02% (best 11.39%) with a *V*_*OC*_ of 0.68 V, a *J*_*SC*_ of 24.94 mA/cm^2^ and FF of 64.97%. Adding 10% of DTBO into the PTB7-Th: IEICO-4F blend dramatically increases the *J*_*SC*_ to 26.43 mA/cm^2^ and FF to 67.64%, resulting in a promising average PCE of 12.48% (best 12.88%). Further addition of DTBO (15 and 20%) led to a decrease in the *J*_*SC*_ and FF. These results show that using a small molecule to form hydrogen bonds with acceptor can dramatically improve the ternary device performance. However, the ternary OSCs based on PTB7-Th: Coumarin 7: IEICO-4F showed a poor performance (Table S1). This result confirms previous predictions that the stronger intermolecular interaction can achieve higher performance in OSCs.

External quantum efficiency (EQE) measurements were conducted to confirm the *J*_*SC*_ of the OSCs. The *J*_*SC*_ calculated from the EQE spectra were coincident with those acquired from *J-V* measurements, with <5% difference between the two methods. As shown in Figure 5B, blending various amounts of DTBO (5–15%), the ternary OSCs show enhancement compared with the binary OSCs, contributing to the increased *J*_*SC*_ value. Notably, due to the enhanced absorption after doping DTBO, the EQE increased from 400 to 500 nm, which means that DTBO is contributing to the photon harvesting charge generation. The ΔEQE (*EQE*_*binary*_−*EQE*_*ternary*_) (Zhao W. et al., 2017; Ma et al., 2018; Song et al., 2019) was calculated to analyze the optimal ternary device. In Figure 5C, the increased ΔEQE from 400 to 600 nm caused by the absorption of DTBO. DTBO absorbed the photons and transferred the energy of the photons to PTB7-Th through Förster resonance process, leading to an increased current. However, the energy transfer only contributes half of increased *J*_*SC*_, the increased EQE from 600 to 1,000 nm also contribute by the better π-π stacking between PTB7-Th and IEICO-4F, which benefit for the charge transfer between donor and acceptor leading an increased EQE from longwave. Moreover, the improved charge carrier collection and reduced recombination contribute to the enhanced EQE values which will be discussed below.

**Figure 6:**
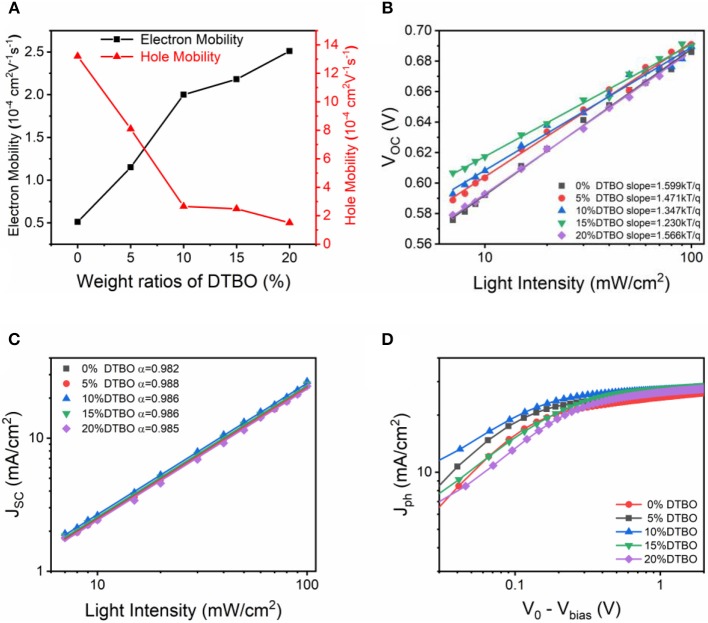
**(A)** Charge mobility of the PTB7-Th: IEICO-4F with different content of DTBO. **(B)** *V*_*OC*_ and **(C)** *J*_*SC*_ dependence on light illumination intensity curves of OSCs devices. **(D)** Photocurrent density (*J*_*ph*_) vs. effective voltage (*V*_0_ − *V*_*bias*_) characteristics.

The charge carrier mobility was measured by using the space-charge-limited-current (SCLC) method (Figure S7) (Mihailetchi et al., 2005). The hole-only devices and electron-only devices were fabricated by using the device architectures ITO/PEDOT: PSS/active layer/MoO_3_/Au and ITO/ZnO/active layer/LiF/Al, respectively. The hole and electron mobility are calculated the slopes of *J*^0.5^*-V* curves by modeling the dark current in the SCLC region. All detailed data are summarized in Figure 6A, Table S2. Notably, for the ternary films, with the increase of DTBO content, electron mobility increased, while hole mobility decreased. The increased electron mobility and the decreased hole mobility contributed to a more balanced charge carrier mobility, leading to a better FF for the device, especially for the device with 10% DTBO content (Bartesaghi et al., 2015).

To understand the charge recombination mechanisms in the devices, the *V*_*OC*_ and *J*_*SC*_ dependence on light intensity was plotted in Figures 6B,C. In general, bimolecular recombination can be investigated by the function of $J_{SC}\propto P_{light}^{\alpha }$, where *P*_*light*_ is the light intensity and α is the exponential factor. If bimolecular recombination is insignificant, the α will close to 1, since all generated excitons are swept out prior to recombination (Kyaw et al., 2013; Lu et al., 2015). The fitting α values are 0.982, 0.988, 0.986, 0.986, and 0.985 for binary OSCs and the ternary OSCs containing 5, 10, 15, and 20% DTBO, suggesting that the bimolecular recombination can be reduced in the optimized ternary OSCs. In the plot of *V*_*OC*_ vs. the natural logarithm of the light intensity, non-geminate recombination can be analyzed by the slope of *nKT/q* (where *k* is Boltzmann constant, *T* is the absolute temperature, and *q* is elementary charge) (Cowan et al., 2010). In general, a slope close to *KT/q* means that the bimolecular recombination is the main reason for the loss mechanism in OSCs, when the trap-assisted recombination is involved, the slope is gradually increased to *2KT/q*. The slope of binary OSCs is 1.599 *KT/q*, by addition of 10% DTBO, the trap-assisted recombination is greatly reduced (1.347 *KT/q*). Which means moderate amount of DTBO can reduce trap-assisted recombination to get a higher FF and better performance in ternary device. Meanwhile, adding more DTBO (>15%) has an adverse effect on suppressing trap-assisted recombination resulting in poor device performance.

The charge generation and extraction properties were analyzed by the photocurrent density (*J*_*ph*_). The *J*_*ph*_ as a function of the effective voltage (*V*_*eff*_) is plotted in Figure 6D. The *J*_*ph*_ is defined as *J*_*ph*_ = *J*_*L*_ − *J*_*D*_, where *J*_*L*_ and *J*_*D*_ are the current density under AM 1.5G and dark current density, respectively. The *V*_*eff*_ is defined as *V*_*eff*_ = *V*_0_ − *V*_*bias*_, where *V*_0_ is the voltage at *J*_*ph*_ = 0 and *V*_*bias*_ is the applied voltage (Blom et al., 2007). Under high *V*_*eff*_ (*V*_*eff*_ > 2*V*), all photon-generated excitons are considered completely dissociated and all photogenerated charge are considered completely extracted by the individual electrode, the saturation current density (*J*_*sat*_) is only limited by the photons harvesting of active layers. In this situation, *J*_*sat*_ can be described as a function of *J*_*sat*_ = *G*_max_*Lq*, where *L* and *q* are the thickness of active layers and elementary charge, *G*_*max*_ is the maximum rate of free charge carrier generation. The calculated *G*_*max*_ of binary and ternary (10, 20%) OSCs are 1.63 × 10^22^, 1.74 × 10^22^, 1.72 × 10^22^ cm^−3^ s^−1^, respectively, which change in the same trend as the *J*_*SC*_ value (Li et al., 2016; Xu et al., 2017). Exciton dissociation efficiency was calculated by *J*_*SC*_/*J*_*sat*_ (Lu et al., 2014). The *J*_*SC*_/*J*_*sat*_ was increased from 91.3 to 95.9% when adding 10% DTBO, suggesting that optimized ternary OSCs has enhanced exciton dissociation. In fact, photocurrent generation also can be limited by charge collection efficiency (η_*coll*_) (Ma et al., 2020b). The high charge collection efficiency can increase the FF and *J*_*SC*_. As for the ternary devices, the incorporation of DTBO also improved the charge collection efficiency. In general, the maximal power output of OSCs usually occurs at the low *V*_*eff*_ regime (<0.5 V). Under the maximal power output condition, all η_*coll*_ values obtained from the optimal ternary device (70.3% at 0.1 V, 86.2% at 0.2 V, and 94.9% at 0.5 V) exceeded those from the binary device (60.2% at 0.1 V, 78.1% at 0.2 V, and 88.8% at 0.5 V) and ternary device with 20% DTBO (48.3% at 0.1 V, 69.9% at 0.2 V, and 88.9% at 0.5 V). This demonstrates the improved charge collection efficiency in the optimal ternary device (10% DTBO) (Liu et al., 2019). These results agree with the increase of *J*_*SC*_ in OSCs devices, proving that addition of 10% of DTBO promotes charge dissociation and extraction, leading to enhanced FF and *J*_*SC*_ for ternary solar cells.

**Figure 7:**
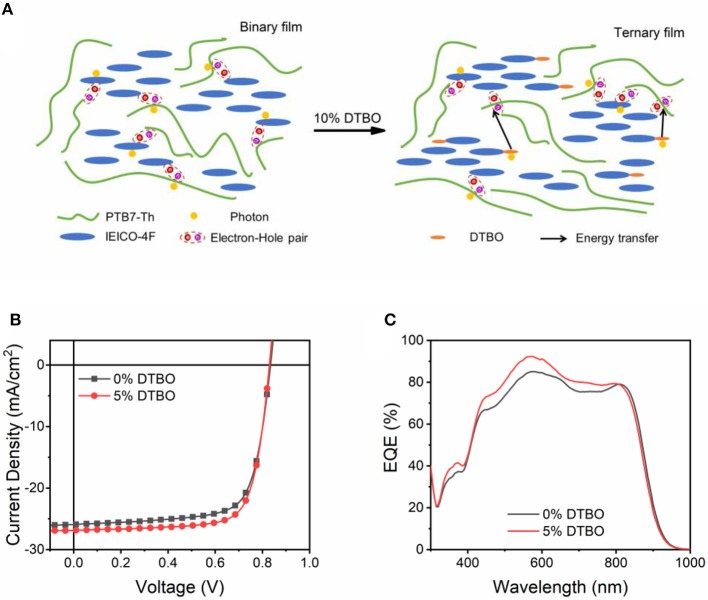
**(A)** Scheme of energy transfer in DTBO based ternary device; **(B)** *J-V* curve of binary and ternary OSCs based PM6: Y6 system; **(C)** EQE curves of OSCs with 0%, 5% DTBO in the PM6: Y6 systems.

Up to now, non-fullerene acceptor with the end group like IEICO-4F show great potential to achieve high PCE. Figure 7A is the scheme of energy transfer in DTBO based ternary device. In order to prove the test this strategy works in other non-fullerene acceptors, we choose the PM6: Y6 as the host system. As shown in Figure S8, the end group of Y6 is the same as that of IEICO-4F, which means the same effect may occur in Y6. The absorption spectra and energy levels of PM6, Y6 are shown in Figure S9. We fabricated devices based inverted architectures, the final PCE increased from 15.49 to 16.64%, and *J*_*SC*_ increased to 26.88 mA/cm^2^ (Table 2). The EQE curves are shown in Figure 7C, which is consist with the change of *J*_*SC*_ (Figure 7B).

**Table 2:** The photovoltaic parameters for PM6: DTBO: Y6 OSCs under AM 1.5 G illumination (100 mW/cm^2^).

**Third compound**	***V_***oc***_*^**a**^ (V)**	***J_***sc***_*^**a**^ (mA/cm^**2**^)**	***J_***calc***_*^**b**^ (mA/cm^**2**^)**	**FF^**a**^ (%)**	**PCE^a^^,^^c^ (%)**
0% DTBO	0.83	25.49	24.52	73.17	15.49 (15.85)
5% DTBO	0.83	26.88	25.59	74.92	16.64 (17.07)

a *All average values were calculated from 10 devices*.

b *J_sc_ integrated from the EQE spectrum*.

c *Best PCE in brackets*.

## Conclusion

In conclusion, we report a novel small molecule DTBO, which could form hydrogen bonds with the acceptor to achieve the increased *J*_*SC*_ and PCE in ternary OSCs. We proved the existence of intermolecular hydrogen bonds between DTBO and IEICO-4F by infrared spectroscopy. The existence of hydrogen bonds can change the ESP of IEICO-4F to promote a better π-π stacking between donor and acceptor which is beneficial for charge transfer. GIWAXS was also used to demonstrate the impact of hydrogen bonds on molecular packing, proving that the hydrogen bonds have a small effect on PTB7-Th, while they have a large effect on IEICO-4F which benefits charge transport. Meanwhile, adding the third component to the binary system not only broadens the absorption spectrum, but also achieves the energy transfer from DTBO to PTB7-Th, leading to a higher *J*_*SC*_. Incorporating DTBO in ternary OSCs also benefits charge generation and extraction, which makes contributions to *J*_*SC*_. The balanced charge carrier mobility in ternary OSCs is the reason why FF increased. In comparison, the ternary OSCs containing Coumarin 7 show poor performance, even though Coumarin 7 and DTBO have a similar structure. Since Coumarin 7 formed a weak hydrogen bond with IEICO-4F, the intermolecular interaction is weaker than that between DTBO and IEICO-4F, resulting in a weak impact on π-π stacking and low PCE. What's more, the strategy is also effective for other non-fullerene acceptors like Y6. Overall, these results may provide a strategy to achieve high OSCs performance by introducing intermolecular hydrogen bond in ternary OSCs to improve the π-π stacking in the active layer, thus achieving better performance.

## Data Availability Statement

All datasets generated for this study are included in the article/Supplementary Material.

## Author Contributions

HZ synthesized DTBO, fabricated, and tested OSCs devices (PTB7-Th:IEICO-4F system). XD designed DTBO and helped design experimental protocols. YT carried out GIWAXS measurements and analysis. XL and LZ fabricated and tested OSCs devices (PM6:Y6 system). CZ and HL calculated ESP with DFT. ST conceived and directed the project. HZ wrote the paper.

### Conflict of Interest

The authors declare that the research was conducted in the absence of any commercial or financial relationships that could be construed as a potential conflict of interest.
