# Supplementary material for: Introducing Trifluoromethyl to Strengthen Hydrogen Bond for High Efficiency Organic Solar Cells
Source: Front Chem. 2020 Mar 24;8:190. doi: 10.3389/fchem.2020.00190 (PMC7105863; doi:10.3389/fchem.2020.00190)
Supplement: Supplementary file 1 [file Data_Sheet_1.PDF]

Supporting Information

A Novel Strategy of Using Hydrogen Bonds to Change the Molecular Stack to Achieve High Efficiency in Organic Solar Cells

Experimental Section

Synthesis

All compounds which is commercially available were used as received without further purification. 4-(dibutylamino)-2-hydroxybenzaldehyde was brought from Aladdin. 4-(trifluoromethyl) benzene-1,2-diamine was brought from Adamas. The DMF, POCl3 were dried and distilled prior to use.

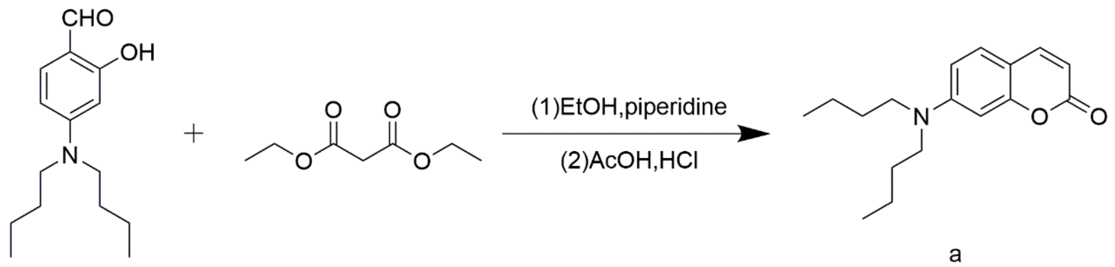

a

Synthesis of 7-(dibutylamino)-2H-chromen-2-one (a)

A mixture of 4-(dibutylamino)-2-hydroxybenzaldehyde (2.5 g, 10 mmol), diethylmalonate (3.2 g, 20 mmol) and piperidine (1 mL) in ethanol (50 ml) was stirred at 75 °C for 6 h under reflux conditions with nitrogen protection. After evaporation of the ethanol, the acetic acid glacial (30 ml) and concentrated HCl (30 ml) were added to the crude solid. The mixture was stirred at 110 °C for 10 h under reflux conditions. After the reaction was cooled to the room temperature, the ice water (200 ml) was added to the solution. 40% solution of NaOH in water was added dropwise to adjusts the PH value to 5, and precipitation occurred at the same time. The precipitation was washed by water. Recrystallization from cyclohexane gave a red brown solid.

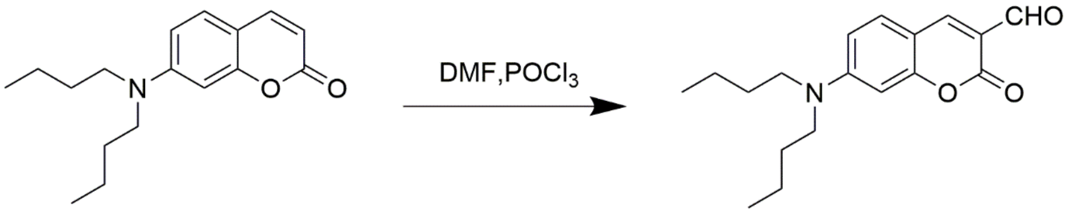

b

Synthesis of 7-(dibutylamino)-2-oxo-2H-chromene-3-carbaldehyde(b)

Anhydrous DMF (3.5 g, 48 mmol) was cooled to 0 ℃ under nitrogen protection, POCl3(3.1 g, 20 mmol) was added dropwise and then the solution was heated at 50 ℃ for 45 min. After cooling to 0 ℃, a solution of compound a (2.2 g, 8 mmol) in DMF (3.5 g, 48 mmol) was added dropwise. Then the mixture was heated at 60 ℃ for 2 h. After cooling, the ice water (100ml) was added to the mixture, and precipitation occurred at the same time. The precipitation was washed by water. Recrystallization from ethanol gave a yellow solid. ^{1}H NMR (300 MHz, Methylene Chloride-d2) δ 10.04 (s, 1H), 8.22 (s, 1H), 7.42 (d, J = 9.0 Hz, 1H), 6.64 (dd, J = 9.1, 2.5 Hz, 1H), 6.48 (d, J = 2.2 Hz, 1H), 3.45 – 3.33 (m, 4H), 1.63 (p, J = 7.6 Hz, 4H), 1.39 (dq, J = 14.6, 7.3 Hz, 4H), 0.98 (t, J = 7.3 Hz, 6H). MS: m/z calculated for $C_{18}H_{23}NO_3$ = 301.17, found 301.16.

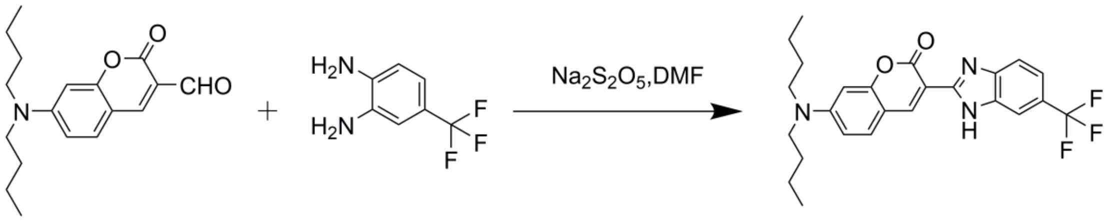

DTBO

7-(dibutylamino)-3-(6-(trifluoromethyl)-1H-benzo[d]imidazol-2-yl)-2H-chromen-2-one (DTBO)

A mixture of compound b (1.5 g, 5 mmol), 4-(trifluoromethyl) benzene-1,2-diamine (0.9 g, 5 mmol) and $Na_2S_2O_5$ (1.9 g 10 mmol) in anhydrous DMF (50 ml) was stirred at 80 °C for 12 h under reflux conditions with nitrogen protection. After cooling, the water (100ml) was added to the mixture. The product was extracted with dichloromethane and washed by water, dried by Na_2SO_4. The solvent removed by rotary evaporation. Then the solid was dried under vacuum at 80 °C. After that the solid was purified by chromatography to give a yellow solid. ^1H NMR (300 MHz, Methylene Chloride-d2) δ 8.94 (s, 1H), 7.57 (s, 1H), 7.51 (d, J = 9.0 Hz, 2H), 7.14 (d, J = 7.5 Hz, 1H), 6.70 (dd, J = 8.9, 2.3 Hz, 1H), 6.55 (d, J = 1.9 Hz, 1H), 3.48 – 3.32 (m, 4H), 1.64 (dt, J = 15.4, 7.7 Hz 4H), 1.40 (dq, J = 14.6, 7.3 Hz, 4H), 0.99 (t, J = 7.3 Hz, 6H). MS: m/z calculated for $C_{25}H_{26}F_3N_3O_2$ = 457.20, found 457.23.

Device fabrication

Active layer materials PTB7-Th and IEICO-4F were purchased from Solarmer Materials Inc. Coumarin 7 and MoO_3 was purchased from Xi'an p-OLED Technology Corp. DTBO was synthesized according to the previous paper. Zinc acetate dihydrate (99.999% trace metals basis) and ethanolamine (purified by redistillation, ⩾99.5%) 2 – Methoxyethanol (anhydrous, 99.8%) were purchased from Sigma-Aldrich Co. All materials were used as received without further purification.

In this work, the inverted structure (ITO/ ZnO/ active layer/ MoO_3/ Ag) was used. The steps of OSCs preparation are as follows. The ZnO layer was prepared by sol-gel precursor method. The method involves dissolving Zinc acetate dihydrate (110 mg) and ethanolamine (31 mg) in 2-methoxyethanol (1 mL), and stirring for eight hours. Spin-

coating was used to produce active layer. The weight ratio of PTB7-Th and IEICO-4F remains 1:1.5 (8 mg/ml for PTB7-Th) dissolving in chlorobenzene (with 4 vol% of 1-chloronaphthalene). Third compound was added to the CB solution with weight of 1:x (1 for PTB7-Th). The solution was stirred at 60 °C for 12 h with nitrogen atmosphere. ITO glass substrates (15 Ω $square^{-1}$) were washed by detergent, ultrasonicated (using DI water, ethanol, acetone, and ethanol), dried by nitrogen, and further processed by O_2 plasma for 30 min. After spin coating of ZnO precursor solution, the substrates were annealed at 200 °C for 1 h in air. After that the active layer solution was spin coated (1500 rpm, 40s) onto the ZnO layer. At last, MoO_3 (10 nm) and Ag (150 nm) were deposited under a pressure of 5×10^{-4} Pa. The active area of device is 0.023 cm^2.

Measurements

1H, 13C NMR spectra of the compounds were recorded on a Bruker AVANCE 300 MHz spectrometer. MS data was measured via Finnigan 4021C gas chromatography mass spectrometry instrument. The FR-IR spectra were tested by a Bruker 80v FT-IR Spectrometer, employing in all cases solid samples (KBr pressed disks). The UV-visible absorption spectra of the active layer were acquired by using a Hitachi U-3010 UV-VS spectrophotometer. The HOMO and LUMO of compounds were measured by a CHI600E Electrochemical Workstation in Tetrabutylammonium hexafluorophosphate dichloromethane solution, and ferrocene/ferrocenium (Fc/Fc+) was used as the external standard. The current density-voltage (*J-V*) was measured in air using a Newport Oriel Sol3A Simulator with AM 1.5 G 100 $mWcm^{-2}$ illumination. The light intensity was calibrated by a standard Si solar cell. The EQE spectra were recorded by the integrated

quantum efficiency measurement system QTEST HIFINITY 5 (Crowntech Inc., USA), which was calibrated with a crystal silicon photovoltaic cell ahead of the measurement. The electron mobility was measured by the SCLC method, and mott-gurney equation J= $\left(\frac{9}{8}\right)\varepsilon_0\varepsilon_r\mu V^2/L^3$ was used to calculate the electron mobility, where ε_o is permittivity of vacuum, ε_r is the relative permittivity of the material, μ is the charge carrier mobility, V is the effective applied voltage, and L is the thickness of the active layer. F-4600 (Hitachi) fluorescence spectrometer was used to collect photoluminescence (PL) spectra data. Grazing-incidence wide-angle X-ray scattering (GIWAXS) was measured at BL18U beam line of shanghai synchrotron radiation facility (SSRF). The scattering signal was recorded using a Pilatus 2M detector. The X-ray light energy is 12.67 keV, and the incidence angle of X-rays was adjusted to 0.05 – 0.12°. All samples are spun on Si substrate.

Characterization

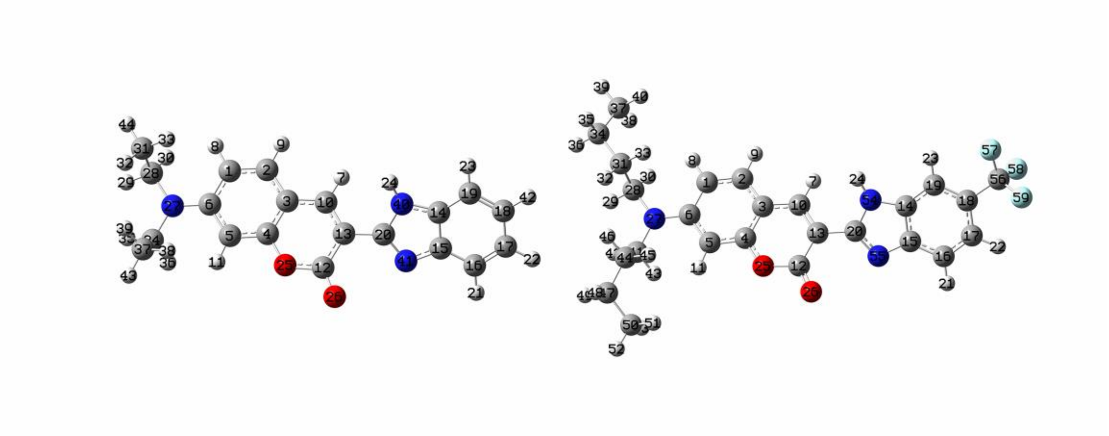

Fig S1. the atom labels of Coumarin 7 and DTBO.

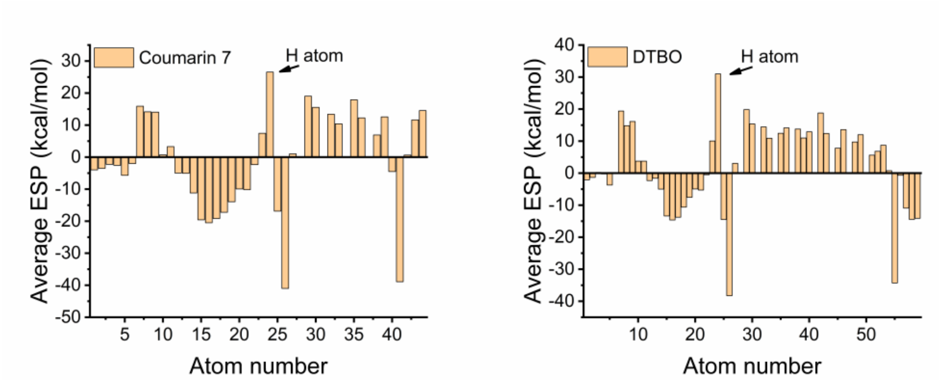

Fig S2. Average ESP statistics of each atom on Coumarin 7 and DTBO.

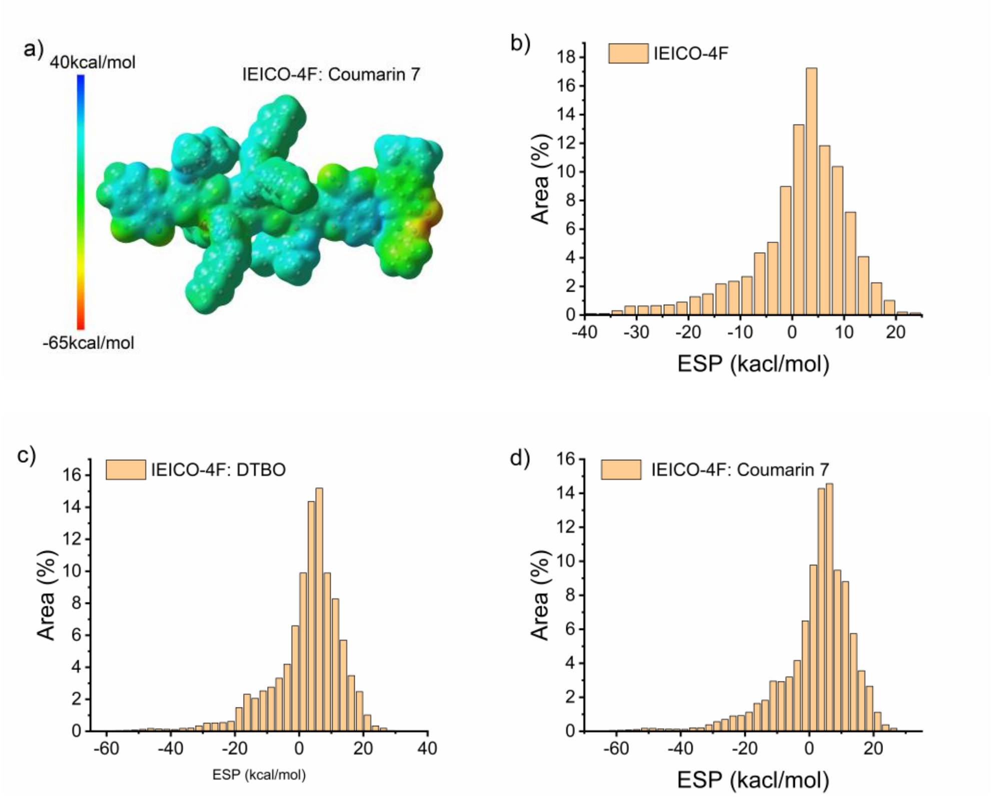

Fig S3. (a) ESP map of hydrogen-bond-linked Coumarin 7: IEICO-4F; ESP area distributions of (b) IEICO-4F (c) DTBO: IEICO-4F (d) Coumarin 7: IEICO-4F.

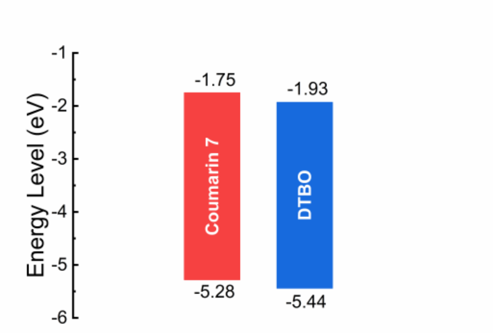

Fig S4. Theoretical calculated HOMO and LUMO of Coumarin 7 and DTBO.

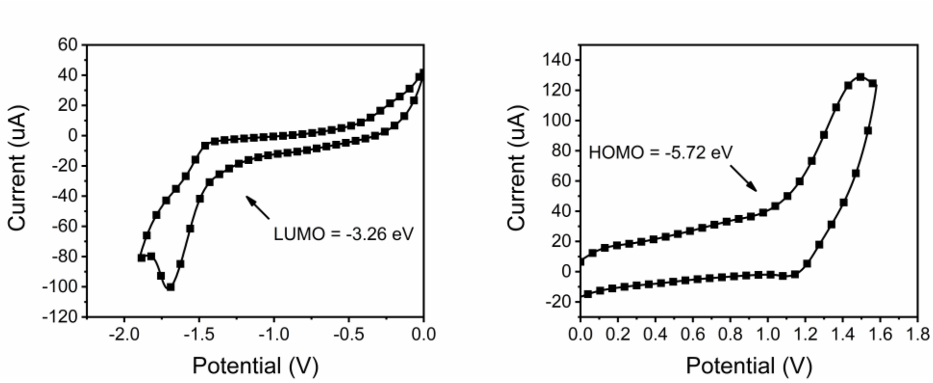

Fig S5. CV plot of DTBO measured in Tetrabutylammonium hexafluorophosphate dichloromethane solution.

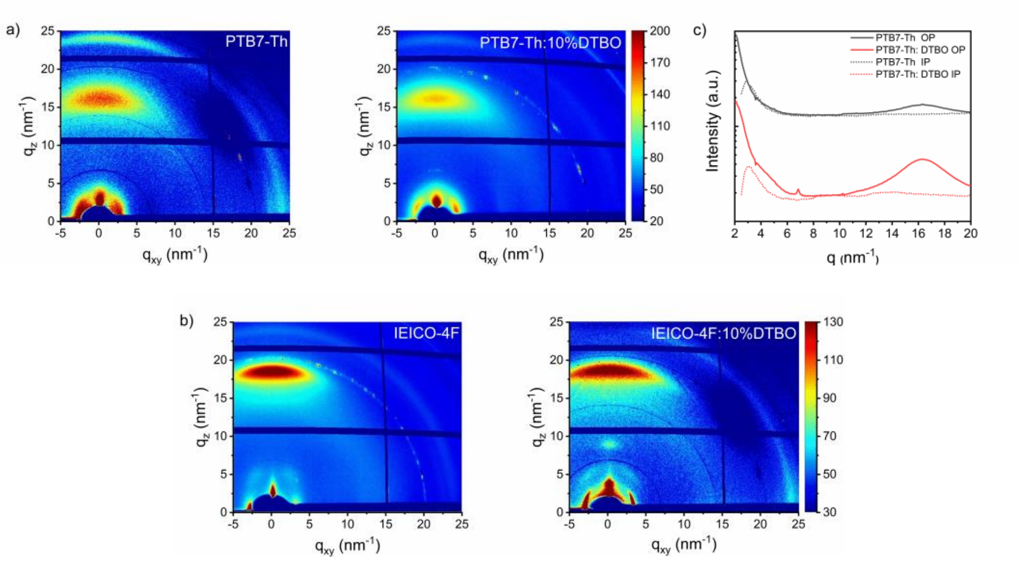

Fig S6. (a) 2-D GIWAXS X-ray scattering pattern of PTB7-Th and PTB7-Th: 10%DTBO (b) line-cut profiles of PTB7-Th and PTB7-Th: 10%DTBO (c) 2-D GIWAXS X-ray scattering pattern of IEICO-4F and IEICO-4F: 10%DTBO.

Table S1. The photovoltaic parameters for PTB7-Th: Coumarin 7: IEICO-4F devices under simulated AM 1.5 G illumination at 100 mW cm^{-2}.

Third compound	V_{oc}^{a} (V)	J_{sc}^{a} (mA/cm^2)	J_{calc}^{b} (mA/cm^2)	FF^{a} (%)	PCE$^{a, c}$ (%)
5% Coumarin 7	0.69	25.28	24.52	68.34	11.87(12.21)
10% Coumarin 7	0.69	26.25	24.91	66.53	12.06(12.33)
15% Coumarin 7	0.71	25.15	24.09	61.57	11.01(11.40)
20% Coumarin 7	0.72	23.99	23.03	61.03	10.52(10.95)

[a] All average values with standard deviations were calculated from 10 devices. [b] J_{sc} integrated from the EQE spectrum. [c] Best PCE in brackets.

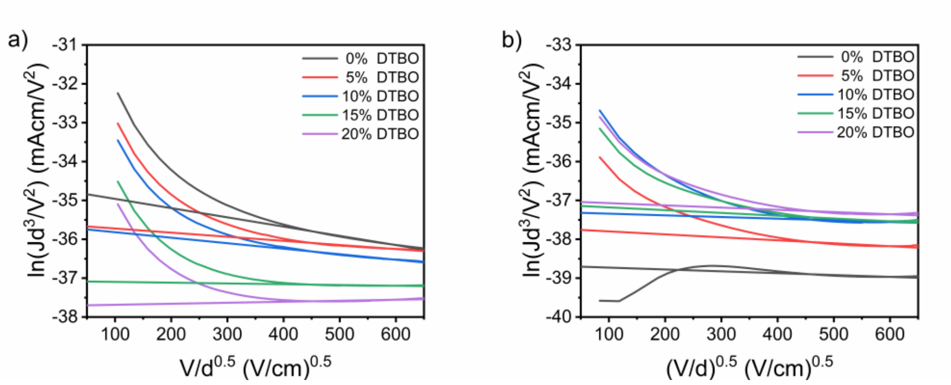

Fig S7. The plotted $ln(Jd^3/V^2)$ versus $(V/d)^{0.5}$ curves of the devices with different DTBO content based (a) hole only devices; (b) electron only devices.

Table S2. The mobility of PTB7-Th: DTBO: IEICO-4F devices.

Third compound	Hole mobility (cm^2 V^{-1} s^{-1})	Electron mobility (cm^2 V^{-1} s^{-1})
0% DTBO	1.32×10^{-3}	5.11×10^{-5}
5% DTBO	8.1×10^{-4}	1.15×10^{-4}
10% DTBO	2.66×10^{-4}	2.00×10^{-4}
15% DTBO	2.49×10^{-4}	2.18×10^{-4}
20% DTBO	1.51×10^{-4}	2.51×10^{-4}

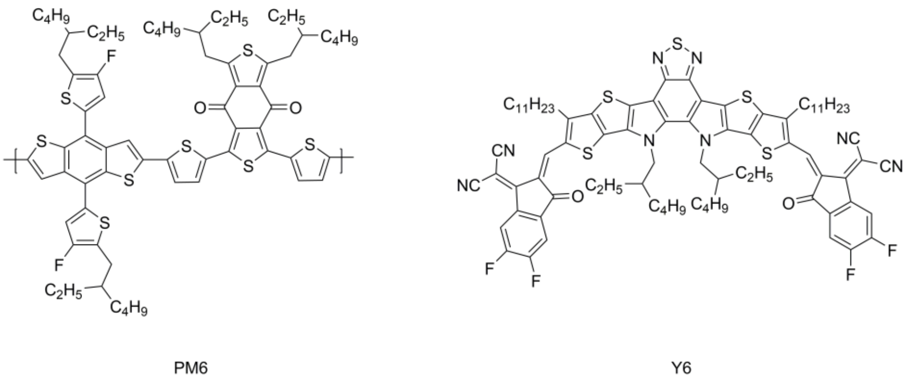

Fig S8. Chemical structure of PM6 and Y6.

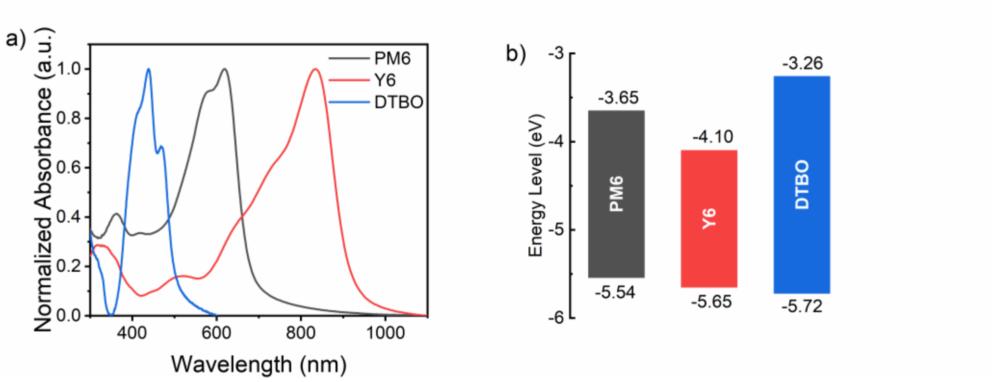

Fig S9. (a)Absorption spectra and (b)energy levels of PM6, Y6 and DTBO.

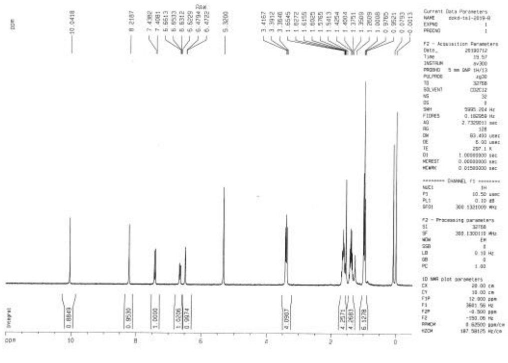

Fig S10. 1H NMR spectra of the intermedia 7-(dibutylamino)-2-oxo-2H-chromene-3-carbaldehyde.

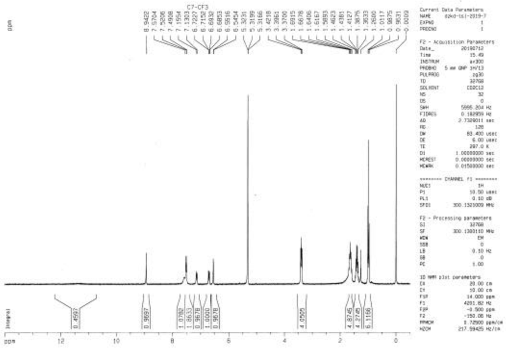

Fig S11. 1H NMR spectra of DTBO.

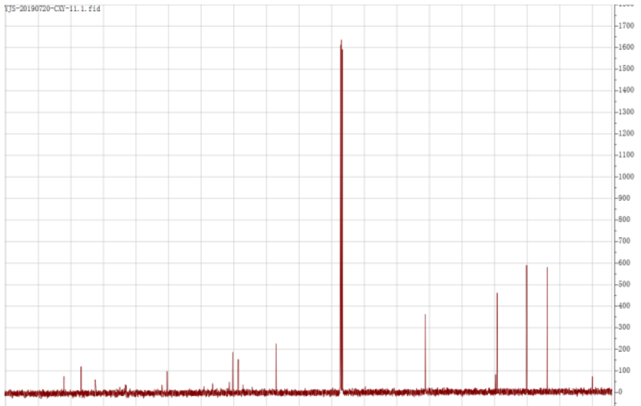

Fig S12. 13C NMR spectra of DTBO.

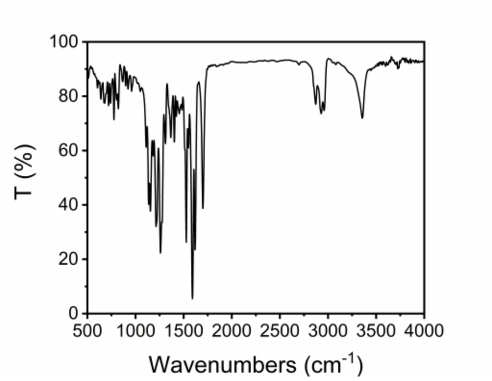

Fig S13. IR spectra of DTBO.
